# Arrhythmogenic Cardiomyopathy as a Late Complication of COVID-19-Induced Myocarditis

**DOI:** 10.7759/cureus.21941

**Published:** 2022-02-05

**Authors:** David Campoamor, Rui Seixas, Leonor Gama, Josiana Duarte, Tiago Araújo

**Affiliations:** 1 Internal Medicine, Unidade Local de Saúde do Litoral Alentejano, Santiago do Cacém, PRT; 2 Internal Medicine, Unidade Local Saúde Litoral Alentejano, Santiago do Cacém, PRT; 3 Internal Medicine, Hospital Litoral Alentejano, Santiago do Cacém, PRT

**Keywords:** ventricular arrhythmia, covid-19, inflammatory cardiomyopathy, arrhythmogenic cardiomyopathy, covid-induced myocarditis

## Abstract

COVID-19 has become a global health problem. So far, more than 281 million COVID-19 cases have been confirmed. The vast majority of patients diagnosed with COVID-19 infection present only with respiratory signs and symptoms. A small amount of patients, however, show signs and symptoms of cardiovascular involvement like a myocardial injury. Myocarditis is one of the possible complications, and cases of clinically suspected myocarditis have been reported in the setting of COVID-19. Herein, we present a case of inflammatory cardiomyopathy, a different type of arrhythmogenic cardiomyopathy, in a 32-year-old man, 40 days after being diagnosed with COVID-19.

## Introduction

Myocarditis is a disease of the myocardium characterized by inflammatory cell infiltration. It can be mediated by a viral infection, especially in developed countries, but can also be induced by other infectious pathogens, such as Borrelia spp., Trypanosoma cruzi, and fungi, as well as toxic substances, drugs, and systemic immune-mediated diseases [1]. In terms of clinical presentation, myocarditis symptoms range from subclinical disease to fatigue, chest pain, heart failure, cardiogenic shock, arrhythmias, and sudden death. With such a variable clinical presentation, a high level of clinical suspicion is needed for proper diagnosis. Patients who exhibit a rise in cardiac biomarkers, such as troponin, changes in electrocardiogram (ECG) suggestive of acute myocardial injury, arrhythmia, or abnormalities in the left ventricular systolic function but an otherwise normal coronary angiogram, should always be suspected for possible myocarditis due to viral infection. A definitive diagnosis of myocarditis is based on identifying changes in endomyocardial biopsy (EMB) and histological (Dallas criteria) and immunohistochemical staining [2]. The Dallas criteria classify the results of EMB into three categories: active myocarditis if light microscopy reveals infiltrating lymphocytes and myocytolysis; borderline or ongoing myocarditis if infiltrating lymphocytes but no myocytolysis is found; and negative for myocarditis if no evidence of infiltrating lymphocytes or myocytolysis is found [2]. In the absence of an EMB, a diagnosis of clinically suspected myocarditis can be made by combining clinical presentation and noninvasive diagnostic findings, such as typical cardiovascular magnetic resonance (CMR) abnormalities [2]. When evidence of cardiac dysfunction and ventricular remodeling in the echocardiogram or CMR evaluation is observed, the condition is defined as inflammatory cardiomyopathy [1]. Treatment of myocarditis includes general nonspecific measures for treating the sequelae of heart disease and arrhythmias according to the prevailing guidelines [2,3]. An implantable cardioverter-defibrillator (ICD) can be beneficial in patients with life-threatening ventricular arrhythmias (VA) after the acute phase of myocarditis, who are receiving optimal medical therapy and have a reasonable expectation of survival with a good functional status for more than one year [4,5]. However, the exact physiopathological mechanisms underlying COVID-19-associated heart disease are so far unknown. SARS-CoV-2 can potentially mediate direct cardiac injury due to angiotensin-converting enzyme 2 (ACE2) tropism, indirectly cause myocarditis via cytokine-mediated cardiotoxicity, or trigger an autoimmune response against heart tissue components [1]. In this case report, the authors present a case of arrhythmogenic cardiomyopathy myocarditis in a young male patient 40 days after SARS-CoV-2 infection.

## Case presentation

A 32-year-old male patient, who was otherwise healthy, presented to the emergency department with acute chest pain and palpitations that started the hour prior to his visit. He tested positive for SARS-CoV-2 infection 40 days earlier and only exhibited dry cough and odynophagia, which resolved after symptomatic treatment with paracetamol. The patient was tachycardic (200 beats per minute), hypotensive (77/41 mmHg), and afebrile, with an oxygen saturation of 98% on room air. ECG showed ventricular tachycardia (VT). Synchronized cardioversion shock at 150 joules was applied with a resolution of the VT to 80 beats per minute of sinus rhythm on ECG re-evaluation. Chest x-ray was normal. Blood samples were collected, which revealed elevated high-sensitivity troponin (149.87 ng/mL). The remaining blood analysis was unremarkable, as shown in Table 1.

**Table 1:** Diagnostic tests of the patient with myocarditis post-SARS-CoV-2 infection.

Parameters	Laboratory Value	Reference Range
White blood cells	7.70	4.00 - 11.00 (x10^9^/L)
Neutrophile cells	4.92	1.60 - 8.30 (x10^9^/L)
Monocytes	0.30	0.20 - 1.10 (x10^9^/L)
Lymphocyte cells	2.30	1.30 - 3.40 (x10^9^/L)
Platelets	175	150 - 400 (x10^9^/L)
Troponin level	149.87	24 - 30 (pg/mL)
NT pro-BNP	70.89	< 125 (pg/mL)
Hemoglobin	13.9	13.5 - 17.0 (g/dL)
C-reactive protein	0.04	0.3 - 10 (mg/dL)
Creatinine	1.1	0.8 - 1.2 (mg/dL)
Sodium (Na)	135	136 - 146 (mmol/L)
Potassium (K)	3.9	3.5 - 5.1 (mmol/L)
Creatine Kinase	178	0.0 - 171 (U/L)
Lactate Dehydrogenase (U/L)	321	208 - 378 (U/L)
D-Dimer (ug/mL)	200	0 - 230 ng/mL
Prothrombin time	13.1	9 - 13 (sec)
Activated partial thromboplastin time	28.8	23.8 - 35.8 (sec)
Thyroid-stimulating hormone	0.59	0.38 - 5.33 (uU/L)
T4 free hormone	1.15	0.61 - 1.13 (ng/dL)

**Figure 1:**
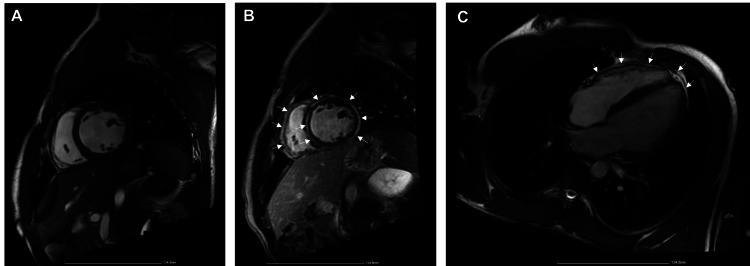
Cardiac magnetic resonance imaging Sagittal plane without contrast (A) showing slightly dilated left ventricle; sagittal plane after contrast injection (B) showing sub-epicardial late enhancement with an annular pattern that progresses through the interventricular septum and extends to the pericardium (C).

Coronary angiography showed no epicardial coronary lesions. The patient was suspected of acute myocarditis and was admitted to an intermediate care unit for close monitoring and further etiological investigation. The nasopharyngeal swab was negative for SARS-CoV-2, as well as for influenza A and B, adenovirus. Serologies for Cytomegalovirus, Epstein-Barr virus, herpes simplex (1, 2, and 6), coxsackievirus, parvovirus B19, hepatitis C, human immunodeficiency virus, and Lyme antibodies, were also negative. Blood tests for autoimmune diseases (smooth muscle antibodies, antineutrophil cytoplasmic antibodies, antinuclear antibodies) and genetic testing for arrhythmogenic cardiomyopathy were negative. Transthoracic echocardiography revealed a dilated left ventricle, mild diffuse hypokinesia with light impairment of global systolic function, and a dilated left atrium. CMR showed extensive subepicardial fibrosis with diffuse contrast uptake at the level of the pericardium. These findings are consistent with myocarditis with pericardial involvement (Figures 1A-1C). Owing to the risk of sudden cardiac death, an ICD was implanted on the 20th day of admission. The procedure was delayed because it had to be performed at another hospital, due to the absence of interventional arrhythmology at our hospital. The patient remained asymptomatic for the rest of the hospitalization period was discharged two days later, and was endorsed for an internal medicine and cardiology consultation. 

## Discussion

COVID-19 usually presents with acute respiratory symptoms but is also known to affect other organs. Pulmonary embolism, cerebral infarction, and acute myocardial infarction are some of the most common cardiovascular diseases associated with SARS-CoV-2 infection. Due to its variable clinical presentation, including subclinical disease, myocarditis often goes undiagnosed; thus, the actual incidence of myocarditis induced by SARS-CoV-2 infection could be higher than currently reported. Cardiac biomarkers such as troponin can detect myocardial injury, but no indications have been established for using troponin as a screening tool in the absence of cardiovascular symptoms.

In the absence of an EMB, CMR, and echocardiography are important diagnostic tools for clinically suspected acute myocarditis induced by SARS-CoV-2 infection. However, they do not quantify the risk of transition from acute myocarditis to chronic cardiomyopathy [1].

Current guidelines on the treatment of VA recommend that ICD implantation for the management of VA should be evaluated after resolving reversible acute myocarditis, generally three to six months after initiation of the acute phase [4,5]. However, the optimal timing remains unclear for several reasons. These guidelines were established based on known viral agents four years before the COVID-19 pandemic. The physiopathological mechanisms underlying SARS-CoV-2-associated heart disease are currently unknown. Furthermore, the risk of sudden cardiac death in patients with acute myocarditis is not always associated with the severity of myocardial inflammation and can persist after the acute phase of myocarditis resolves [6].

The reported cases of cardiomyopathy associated with SARS-CoV-2-associated myocarditis are referred to as stress-induced (Takotsubo) cardiomyopathy. The case we present is an example of inflammatory arrhythmogenic cardiomyopathy, and as far as the authors know, is one of the first cases of clinically suspected cardiomyopathy associated with COVID-19, since there was no histological confirmation [7]. In this case, an ICD was implanted before the resolution of acute myocarditis because sudden cardiac death was a concern.

## Conclusions

As myocarditis induced by SARS-CoV-2 infection can evolve to chronic cardiomyopathy, all efforts must be made for accurate diagnosis. Further investigation on the physiopathological mechanisms underlying SARS-CoV-2-associated heart disease, as well as patterns in CMR and ECG changes, are needed to predict and prevent the risk of transition from acute myocarditis to chronic cardiomyopathy. This information is also crucial in developing specific recommendations on arrhythmia management.
